# Age-Sensitive Usability in Conversational AI Agents: A Systematic Review

**DOI:** 10.1093/geroni/igaf122.3533

**Published:** 2025-12-31

**Authors:** Agnes Jihae Kim, Moon Choi

**Affiliations:** Korea Advanced Institute of Science and Technology, Daejeon, Republic of Korea; Korea Advanced Institute of Science and Technology, Daejeon, Republic of Korea

## Abstract

The rapid rise of conversational AI agents, exemplified by ChatGPT, has shaped daily life from health information seeking to emotional support. While offering opportunities and challenges for aging populations, most designs remain youth-centric. This systematic review critically assesses existing knowledge on usability evaluation of conversational AI agents for older adults. Following the PRISMA-ScR guidelines, four major databases (Web of Science, Scopus, ACM Digital Library, and PubMed) were searched using the keywords “older adults,” “conversational AI agents,” and “usability.” After removing duplicates, 873 journal articles were identified. Screening through Covidence software yielded 15 studies that met the inclusion criteria by targeting or separately reporting on older adults. Analysis showed that healthcare (42.9%) and independent living activities (33.3%) were the primary domains of application, while companion support (14.3%) and social care (9.5%) were less frequently studied but emerging. Most studies were published after 2020, with nine conducted in Europe and the U.S. and five in Asia. Regarding measurement, the System Usability Scale (SUS) and satisfaction surveys were most commonly applied, while AI-specific measures (e.g., the Chatbot Usability Scale) were rarely used. Across the seven studies employing SUS, the average score was 70.6, exceeding the benchmark of 68 points and suggesting generally acceptable usability. This review synthesizes current evidence on usability evaluation of conversational AI agents among older adults, who often present distinct cognitive, physical, and behavioral characteristics related to digital technology use. It underscores the need for age-sensitive design and evaluation frameworks to move beyond trial-and-error practices and inform inclusive AI policies.

